# Distribution of *Malassezia* species on the skin of patients with atopic dermatitis, psoriasis, and healthy volunteers assessed by conventional and molecular identification methods

**DOI:** 10.1186/1471-5945-14-3

**Published:** 2014-03-07

**Authors:** Tomasz Jagielski, Elżbieta Rup, Aleksandra Ziółkowska, Katarzyna Roeske, Anna B Macura, Jacek Bielecki

**Affiliations:** 1 Department of Applied Microbiology, Institute of Microbiology, Faculty of Biology, University of Warsaw, I. Miecznikowa 1, 02-096 Warsaw, Poland; 2 Department of Mycology, Chair of Microbiology, Collegium Medicum, Jagiellonian University, Cracow, Poland

**Keywords:** Identification, *Malassezia* spp, PCR-RFLP, Sequence analysis, Drug susceptibility

## Abstract

**Background:**

The *Malassezia* yeasts which belong to the physiological microflora of human skin have also been implicated in several dermatological disorders, including pityriasis versicolor (PV), atopic dermatitis (AD), and psoriasis (PS). The *Malassezia* genus has repeatedly been revised and it now accommodates 14 species, all but one being lipid-dependent species. The traditional, phenotype-based identification schemes of *Malassezia* species are fraught with interpretative ambiguities and inconsistencies, and are thus increasingly being supplemented or replaced by DNA typing methods. The aim of this study was to explore the species composition of *Malassezia* microflora on the skin of healthy volunteers and patients with AD and PS.

**Methods:**

Species characterization was performed by conventional, culture-based methods and subsequently molecular techniques: PCR-RFLP and sequencing of the internal transcribed spacer (ITS) 1/2 regions and the D1/D2 domains of the 26S rRNA gene. The Chi-square test and Fisher’s exact test were used for statistical analysis.

**Results:**

*Malassezia sympodialis* was the predominant species, having been cultured from 29 (82.9%) skin samples collected from 17 out of 18 subjects under the study. Whereas AD patients yielded exclusively *M. sympodialis* isolates, *M. furfur* isolates were observed only in PS patients. The isolation of *M. sympodialis* was statistically more frequent among AD patients and healthy volunteers than among PS patients (*P <* 0.03). Whether this mirrors any predilection of particular *Malassezia* species for certain clinical conditions needs to be further evaluated. The overall concordance between phenotypic and molecular methods was quite high (65%), with the discordant results being rather due to the presence of multiple species in a single culture (co-colonization) than true misidentification. All *Malassezia* isolates were susceptible to cyclopiroxolamine and azole drugs, with *M. furfur* isolates being somewhat more drug tolerant than other *Malassezi*a species.

**Conclusions:**

This study provides an important insight into the species composition of *Malassezia* microbiota in human skin. The predominance of *M. sympodialis* in both normal and pathologic skin, contrasts with other European countries, reporting *M. globosa* and *M. restricta* as the most frequently isolated *Malassezia* species.

## Background

The fungi of the *Malassezia* genus, whose first description dates back to the middle of the XIX century, have only recently gained considerable attention of the dermatological community. This is because those basidiomycetous yeasts, being components of the microbiota of human and animal skin and constituting up to 80% of the total skin fungal population [1] are now increasingly recognized as opportunistic pathogens resulting in different dermatological pathologies. *Malassezia* yeasts are the causative agents of pityriasis versicolor (PV), which is one of the commonest superficial mycoses in human population worldwide [2]. *Malassezia* species are also involved in the pathogenesis of various dermatoses with global distribution, such as seborrheic dermatitis (SD), atopic dermatitis (AD), and, more recently, psoriasis (PS) [3-6]. A growing number of reports demonstrate the implication of *Malassezia* yeasts in other skin disorders, including folliculitis, onychomycosis, confluent and reticulated papillomatosis, and neonatal cephalic pustulosis [7-10]. Finally, *Malassezia* yeasts have been associated with systemic infections and outbreaks in neonatal and immunocompromised adults intensive care units [11,12].

The species content of the *Malassezia* genus has repeatedly been revised over the last two decades. In the early 1990s the genus *Malassezia* contained only three species, namely *M. furfur*, *M. pachydermatis*, and *M. sympodialis*. In 1996, four other species (i. e. *M. globosa*, *M. obtusa*, *M. restricta*, and *M. slooffiae*) were identified within the genus, through a comparative sequence analysis of nuclear ribosomal DNA (rDNA) operons [13]. Since 2002 seven more new species have been described, on the basis of molecular data: *M. dermatis*, *M. equina*, *M. japonica*, *M. nana*, *M. yamatoensis*, *M. caprae*, and *M. cuniculi*[14-19]. Overall, the genus *Malassezia* now accommodates 14 species, all but one (*M. pachydermatis*) being lipid-dependent species.

Among the *Malassezia* species, one of the most frequently isolated is *M. sympodialis*, which has been considered to be associated with AD. One of the clearest indications to support this is the fact that almost half of the adult patients suffering from AD are sensitized to *M. sympodialis*, as evidenced by allergen-specific IgE and/or T-cell reactivity to the yeast, and that such reactivity is rarely observed in other allergic diseases [20,21].

The identification of most of *Malassezia* species can be achieved by using a combination of morphological, biochemical, and physiological characteristics. The currently used protocol, based on the phenotypic criteria, includes the examination of colony and cell morphology, determination of urease, catalase, and β-glucosidase activities, growth at 37°C and 40°C, and the capacity to grow with different polyoxyethylene sorbate compounds (Tween 20, 40, 60, 80) and polyoxyethylene castor oil (Cremophor EL), as the sole lipid source [22].

With the advent of molecular biology tools, several new, DNA-targeted methods have been developed and tested for species identification within the *Malassezia* taxon. These include pulsed-field gel electrophoresis (PFGE) [23,24], randomly amplified polymorphic DNA (RAPD) analysis [23,25], amplified fragment length polymorphism (AFLP) analysis [25,26], denaturing gradient gel electrophoresis (DGGE) [25], multilocus enzyme electrophoresis (MEE) [27], PCR-based single strand confirmation polymorphism (PCR-SSCP), PCR-based restriction fragment length polymorphism (PCR-RFLP) [7,28-34], nested PCR [32,35-37], real-time (RT) PCR [35,38], and direct sequencing of various genetic loci, such as rDNA and internal transcribed spacer (ITS) 1 and 2 regions in particular, the chitin synthase (*CHS2*) gene, and the RNA polymerase subunit 1 (*RPB1*) gene [18,26,39-41].

The purpose of this study was to explore the species composition of *Malassezia* microflora on the skin of healthy volunteers and patients with AD and PS. Species determination was performed by using both conventional, culture-based methods and molecular techniques, that is PCR-RFLP and direct sequencing targeting the rDNA cluster of the *Malassezia* genome.

## Methods

### Subjects

The study group comprised of 18 subjects (8 (44.4%) males and 10 (55.6%) females, aged from 22–70 years; mean age: 34.8 years; median age: 30 years), all being Polish and living in the Lesser Poland province. The subjects were split into three, equally sized groups (i.e. six subjects per each group), consisting of healthy volunteers (i) and patients diagnosed with either atopic dermatitis (ii) or psoriasis (iii). Both AD and PS patients were recruited from the routine dermatology outpatient clinic at the Collegium Medicum of the Jagiellonian University in Kraków, where they were assessed clinically and received treatment during a 3-year period (2008–2010). The diagnosis of AD was made according to the Hanifin-Rajka criteria [42], and the severity of the disease was categorized based on the Severity Scoring of Atopic Dermatitis (SCORAD) index [43]. The diagnosis of PS was based on clinical features and confirmed by histopathological analysis. The severity of psoriatic lesions was evaluated using the Body Surface Area (BSA) and Psoriasis Area and Severity (PASI) indexes [44,45].

Information on demographic characteristics and clinical aspects of the disease were collected in a standardized questionnaire by reviewing the medical records and analysed (Table 1).

**Table 1:** **Characteristics of 18 subjects under the study and microbiological details of 35 isolated *Malassezia *** **species**

	**Patient**^***a***^	**Subject group**^***b***^	**Collection site**	**Sex**^***c***^	**Age**	**Lesion severity**^***d***^	**Episode**^**e**^	**Duration of disease [yrs]**	**Treatment**^***f***^	**Family history of disease**	**Other diseases**^***g***^	**Strain no.**	**Species identifiction**^***h***^
**1.**	TK	AD	Head	M	24	37	R	24	Topical; sys: antihistaminics	Positive	No	40.10.I.	*M. sympodialis*
**2.**			Face									40.10.II.	*M. sympodialis*
**3.**			Back									40.10.IV.	*M. sympodialis*
**4.**	MP		Head	F	28	31	R	23	Topical; sys: antihistaminics	Positive	Rhinoconjunctivitis	8.11.I.	*M. sympodialis*
**5.**			Chest									8.11.III.	*M. sympodialis* + C. d.^*^
**6.**			Back									8.11.IV.	*M. sympodialis*
**7.**	DJ		Chest	M	29	34.6	R	29	Topical; sys: antihistaminics	Positive	Rhinoconjunctivitis	27.10.III.	*M. sympodialis*
**8.**			Back									27.10.IV.	*M. sympodialis*
**9.**	JW		Chest	F	22	10.2	R	22	Topical; sys: antihistaminics	Positive	No	7.11.III.	*M. sympodialis*
**10.**			Back									7.11.IV.	*M. sympodialis*
**11.**	MB		Back	M	22	39.4	R	22	Topical; sys: antihistaminics	Negative		25.10.IV.	*M. sympodialis*
**12.**	EP		Back	F	31	10.2	R	31	Topical; sys: antihistaminics	Negative	Vitiligo	17.10.IV.	*M. sympodialis*
**13.**	BT	PS	Head	F	50	8.2	R	20	Topical; sys: CyA	Positive	No	10.11.I.	*M. sympodialis*
**14.**			Face									10.11.II.	*M. sympodialis* + A. p.^**^
**15.**			Chest									10.11.IIIA.	*M. furfur*
**16.**			Back									10.11.IV.	*M. sympodialis*
**17.**	BH		Face	F	44	10	R	15	Topical	Negative	No	45.08.II.	*M. furfur*
**18.**			Chest									45.08.III.	*M. furfur* + *M. sympodialis*
**19.**	SK		Back	M	38	3.6	R	5	Topical; sys: CS	Negative	No	20.09.IV.	*M. furfur* + *M. sympodialis*
**20.**	EN		Back	F	31	6.6	R	5	Topical	Negative	No	17.09.IV.	*M. sympodialis*
**21.**	KK		Face	M	23	11.2	R	12	Topical; sys: CyA	Positive	No	43.08.II.	*M. furfur*
**22.**	MB		Back	F	29	5.7	R	15	Topical	Positive	No	6.11.IV.	*M. sympodialis*
**23.**	MT	Control	Head	F	31						No	1.11.IA.	*M. globosa*
**24.**			Face									1.11.II.	*M. globosa* + *M. restricta*
**25.**			Chest									1.11.IIIA.	*M. sympodialis*
**26.**			Back									1.11.IVA.	*M. sympodialis*
**27.**	CM		Chest	M	70						No	69.09.III.	*M. slooffiae*
**28.**			Back									69.09.IV.	*M. slooffiae* + *M. sympodialis*
**29.**	BP		Chest	M	69						No	67.09.III.	*M. sympodialis*
**30.**			Back									67.09.IV.	*M. sympodialis*
**31.**	PK		Chest	M	27						No	68.09.III.	*M. sympodialis*
**32.**			Back									68.09.IV.	*M. sympodialis*
**33.**	MS		Chest	F	28						No	2.11.III.	*M. sympodialis*
**34.**			Back									2.11.IV.	*M. sympodialis*
**35.**	JP		Back	F	31						No	12.09.IV.	*M. sympodialis*

^*a*^Patient initials.

^*b*^Three subject groups under investigation: (i) patients with atopic dermatitis (AD), (ii) patients with psoriasis (PS), and (iii) healthy volunteers (control).

^*c*^M, male; F, female.

^*d*^The numbers represent the SCORAD index values in AD patients, or the PASI index values in psoriatic patients.

^*e*^R, recurrent.

^*f*^Topic treatment for AD patients included emolients, corticosteroids, tacrolimus, pimecrolimus, antibiotics, whereas topic treatment for psoriatic patients included corticosteroids, calcipotriol, tacrolimus, pimecrolimus, emollients; CS, corticosteroids; CyA: Cyclosporine A; sys., systemic.

^*g*^Based on direct interviewing of the patients.

^*h*^Final species identification results, based upon integration of conventional and molecular data. ^*^C. d., *Cryptococcus diffluens*; ^**^A. p., *Aureobasidium pullulans*.

### Sample collection

From each patient, four samples originating from four different anatomical sites of the body, that is the scalp, face, chest (interclavicle region) and back (interscapular region) were collected by a standard swab method. A sterile cotton swab soaked with sterile saline was used to rub against the skin surface, with continuous rotation of the swab and over at least 15 seconds, and immediately streaked evenly onto modified Dixon’s agar (mDA) medium.

### Culture conditions and yeast strains

The yeasts were cultured on mDA plates at 32°C, with growth being monitored every day for two weeks. The suspected colonies of *Malassezia* sp. were subcultured, by streaking onto mDA slants, and subjected to the identification procedures described hereafter. The cultures were maintained by weekly passaging on fresh mDA slants.

Apart from the *Malassezia* sp. strains isolated from clinical samples, six reference strains representing as many *Malassezia* species (*Malassezia furfur* CBS 6001; *M. globosa* CBS 7966; *M. obtusa* CBS 7876; *M. slooffiae* CBS 7956; *M. restricta* CBS 7877; *M. sympodialis* CBS 7222) and purchased from the CBS (Centraalbureau voor Schimmelcultures, Utrecht, The Netherlands) culture collection were included in the study.

### Drug susceptibility testing

*In vitro* susceptibility testing was performed by using commercially available Neo-Sensitabs diffusion assays (Neo-Sensitabs, Rosco Diagnostica, Denmark), according to the instructions provided by the manufacturer and following the Clinical and Laboratory Standards Institute (CLSI) guidelines [46] with some modifications. Colonies from a seven-day yeast culture, grown on mDA, were scraped off and suspended in sterile saline with sterile glass beads. The suspension was mixed vigorously for 30 seconds in a vortex mixer, and adjusted to a turbidity equivalent of a 5.0 McFarland standard. The so prepared inoculum was swabbed over the mDA plates. After allowing the plates to dry completely, Neo-Sensitabs tablets containing 50 *μ*g of ciclopirox (CPO), 25 *μ*g of fluconazole (FLZ), 15 *μ*g of ketoconazole (KTZ), 10 *μ*g of econazole (ECZ), 10 *μ*g of miconazole (MNZ), and 8 *μ*g of itraconazole (ITZ) were applied onto the surface and the plates were incubated at 32°C, with reading taken after 48 and 72 h. The zones of inhibition were measured at a point in which either there was prominent reduction of growth or no visible growth occurred. Since the interpretation of antifungal resistant/susceptible categories among the *Malassezia* species has not yet been established, interpretive criteria for the yeasts reported by the CLSI [46] and provided by the manufacturer of the Neo-Sensitabs were employed. Accordingly, resistance was assumed if the inhibition zone was less than 10 mm for ITZ, less than or equal to 11 mm for CPO, ECZ, and MNZ, less than or equal to 14 mm for FLZ, and less than or equal to 22 mm for KTZ.

To validate the performance of the Neo-Sensitabs drug susceptibility testing, two quality control (QC) strains were used: *Pichia kudriavzevii* (teleomorph *of Candida krusei*) DBVPG 7235 (corresponding to ATCC 6258) and *Candida parapsilosis* DBVPG 6150 (corresponding to ATCC 22019), from the Industrial Yeasts Collection DBVPG (Perugia, Italy).

All *Malassezia* strains were tested in duplicate. Also, the QC strains were assayed twice.

### DNA isolation

Genomic DNA extraction was done from pure cultures. Briefly, a few yeast colonies were suspended in 200 *μ*L of TE buffer (10 mM Tris–HCl [pH 8.0], 1 mM EDTA) and subjected to 3 rounds of sonication (three sonication cycles of 15 s each separated by 15 s intervals) in ice water bath, at 20% amplitude in a Vibra Cell sonicator (Sonics & Materials Inc., USA). The obtained homogenate was further processed with the Genomic Mini AX Yeast kit (A&A Biotechnology, Poland) following the manufacturer’s instructions.

### Species identification

#### Identification by phenotypic methods

All yeast cultures were identified to species level by using conventional mycological methods, including examination of colonial and microscopic morphologies (i.e. colonies’ shape, texture, and colour, and cell size, shape and budding characteristics) as well as physiological and biochemical tests, including assimilation of Tween 20, 40, 60, and 80, assimilation of Cremophor EL, catalase reaction, cleavage of esculin, and growth at 37°C. Phenotypic feature testing was performed essentially as described elsewhere [47], and the characteristics table of *Malassezia* spp. provided by Ashbee and Evans [48] served as the species identification key.

#### Molecular analysis

Molecular identification involved PCR-RFLP analysis along with sequence analysis of different nuclear loci within the rDNA operon of *Malassezia* spp., including the ITS1 and ITS2 regions and the D1 and D2 domains of the 26S rRNA gene.

#### PCR-RFLP analysis

**Figure 1:**
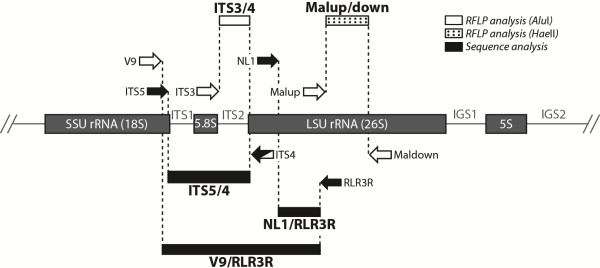
**Schematic representation of the rDNA operon in *Malassezia *yeasts.** The loci under analysis are depicted. Major rRNA genes are shown as grey boxes. Primers used for PCR amplification of the target DNA sequences and those used for sequencing are indicated as white and black block arrows, respectively. Loci analysed by PCR-RFLP or direct sequencing are shown as black or white rectangles, respectively, and are designated after the primers used for their amplification (or sequencing). LSU, large subunit (rRNA); SSU, small subunit (rRNA); IGS, intergenic spacer region; ITS, internal transcribed spacer.

Two PCR-RFLP assays, targeting the ITS2 region and partial 26S rRNA gene, were performed, as described previously [28,29], with slight modifications. Briefly, primers ITS3 (5′-GCATCGATGAAGAACGCAGC-3′) and ITS4 (5′-TCCTCCGCTTATTGATATGC-3′) were used to amplify the ITS2 region, whereas primers Malup (5′-AGCGGAGGAAAAGAAACT-3′) and Maldown (5′-GCGCGAAGGTGTCCGAAG-3′) were used to amplify the 26S rRNA gene fragment (Figure 1). PCR mixtures were prepared by using the TopTaq Master Mix kit (QIAGEN, Germany) in a total volume of 25 *μ*L, containing 2× TopTaq Master Mix (final conc. 1×; the mix contains 1.25 U of *Taq* DNA polymerase, 200 *μ*M each deoxyucleoside triphosphate, and 1× PCR buffer with 1.5 mM MgCl_2_), 0.4 *μ*M each primer, and 1 *μ*L (*ca*. 10–20 ng) of template DNA. The PCR conditions were as follows: 94°C for 3 min, 30 cycles at 94°C for 30 s, 50°C for 30 s, and 72°C for either 30 s (ITS2) or 50 s (26S rDNA), and a final step at 72°C for 10 min. After confirmation of the presence of the amplicons of correct sizes with gel electrophoresis, the purified PCR products of the ITS2 and 26S rDNA regions were subjected to RFLP analysis with *Alu*I and *Hae*II enzymes, respectively. Restriction reactions were carried out in 20 *μ*L volumes containing 8 *μ*L (*ca*. 200 ng) of PCR product and 1 U of the restriction enzyme together with the appropriate reaction buffer (final conc. 1×) at 37°C for 45 min (FastDigest restriction enzymes; Fermentas UAB, Lithuania). Restriction patterns were compared with those of reference *Malassezia* strains and virtual, species-specific patterns established in the original publications [28,29].

#### PCR-sequencing

Sequencing of the ITS1 and ITS2 regions and the D1/D2 domains of the 26S rRNA gene was performed, as reported earlier [26]. In short, primers V9 (5′-TGCGTTGATTACGTCCCTGC-3′) and RLR3R (5′-GGTCCGTGTTTCAAGAC-3′) amplified a fragment encompassing ITS1, the 5.8S rRNA gene, ITS2, and partial regions of the 18S and 26S rDNA (Figure 1).

The PCR was carried out in a 25-*μ*L reaction volume containing 2× TopTaq Master Mix (final conc. 1×) (QIAGEN, Germany), 0.5 *μ*M each primer, and 1 *μ*L (*ca*. 20 ng) of template DNA. The thermal cycling profile was 94°C for 5 min, 30 cycles at 94°C for 45 s, 56°C for 30 s, and 72°C for 1.5 min, and a final step at 72°C for 10 min. The resulting amplicons were electrophoresed to verify the presence of a single product of the correct size, purified, and sequenced either directly or after cloning into a plasmid vector using the pGEM-T Easy Vector system (Promega, USA), according to the vendor’s protocol, when initial sequence information showed the possible presence of two (or more) different species.

The primers used in the sequence reaction were ITS4 (5′-TCCTCCGCTTATTGATATGC-3′) and ITS5 (5′-GGAAGTAAAAGTCGTAACAAGG-3′) for analysis of the ITS1/ITS2, and NL1 (5′-GCATATCAATAAGCGGAGGAAAAG-3′) and RLR3R for analysis of the D1/D2 domains of the 26S rRNA gene (Figure 1).

Forward and reverse sequences were assembled and edited with ChromasPro ver. 1.7.1 (Technelysium, Australia) and the resulting consensus sequences were searched against the GenBank database of the National Center for Biotechnology Information (NCBI) using the BLASTN algorithm (http://blast.ncbi.nlm.nih.gov/).

Distance scores of up to 1.00% (99% match) were used as a proxy for species identity, and the species giving the closest match was considered the correct identification.

The nucleotide sequences determined in the study were deposited in the GenBank database (NCBI) under the accession numbers listed in Table 2.

**Table 2:** **Species identification results obtained upon phenotypic testing and molecular typing of 35 *Malassezia *** **cultures**

	**Strain no.**	**Species identification by means of**								**Final species identification**^***d***^
		**Phenotypic methods**^***a***^	**Molecular methods**							
			**PCR-RFLP**^***b***^	**PCR sequencing**^***c***^						
				**ITS accession no.**	**Similarity with ITS of [%]:**		**D1/D2 accession no.**	**Similarity with D1/D2 of [%]:**		
**1.**	40.10.I.	*M. slooffiae*	*M. sympodialis*	KC141968	*M. sympodialis* MA 73	100%	KC415092	*M. sympodialis* IFM 48109	100%	*M. sympodialis*
**2.**	40.10.II.	*M. slooffiae*	*M. sympodialis*	KC141969	*M. sympodialis* CBS 7222	100%	KC415093	*M. sympodialis* IFM 48109	100%	*M. sympodialis*
**3.**	40.10.IV.	*M. sympodialis*	*M. sympodialis*	KC141970	*M. sympodialis* CBS 7222	100%	KC415094	*M. sympodialis* IFM 48109	100%	*M. sympodialis*
**4.**	8.11.I.	*M. obtusa*	*M. sympodialis*	KC152895	*M. sympodialis* CBS 7222	100%	KC415085	*M. sympodialis* IFM 48109	100%	*M. sympodialis*
**5.**	8.11.III.	*M. furfur*	*M. sympodialis*	KC152904	*C. diffluens* CBS 160	100%	KC241877	*C. diffluens* CBS 6436	100%	*M. sympodialis* + C. d.^*^
**6.**	8.11.IV.	*M. sympodialis*	*M. sympodialis*	KC152896	*M. sympodialis* CBS 7222	100%	KC415086	*M. sympodialis* IFM 48109	100%	*M. sympodialis*
**7.**	27.10.III.	*M. sympodialis*	*M. sympodialis*	KC141966	*M. sympodialis* MA 73	100%	KC415095	*M. sympodialis* IFM 48109	100%	*M. sympodialis*
**8.**	27.10.IV.	*M. sympodialis*	*M. sympodialis*	KC141967	*M. sympodialis* MA 73	100%	KC415096	*M. sympodialis* IFM 48109	100%	*M. sympodialis*
**9.**	7.11.III.	*M. sympodialis*	*M. sympodialis*	KC152893	*M. sympodialis* CBS 7222	100%	KC415083	*M. sympodialis* IFM 48109	100%	*M. sympodialis*
**10.**	7.11.IV.	*M. sympodialis*	*M. sympodialis*	KC152894	*M. sympodialis* MA 73	100%	KC415084	*M. sympodialis* IFM 48109	100%	*M. sympodialis*
**11.**	25.10.IV.	*M. sympodialis*	*M. sympodialis*	KC119577	*M. sympodialis* CBS 7222	100%	KC415097	*M. sympodialis* IFM 48109	100%	*M. sympodialis*
**12.**	17.10.IV.	*M. sympodialis*	*M. sympodialis*	KC119576	*M. sympodialis* CBS 7222	100%	KC415098	*M. sympodialis* IFM 48109	100%	*M. sympodialis*
**13.**	10.11.I.	*M. sympodialis*	*M. sympodialis*	KC152897	*M. sympodialis* CBS 7222	100%	KC415087	*M. sympodialis* IFM 48109	100%	*M. sympodialis*
**14.**	10.11.II.	*M. sympodialis*	*M. sympodialis*	KC152905	*A. pullulans* CPC 13701	100%	KC241878	*A. pullulans* RA406	100%	*M. sympodialis* + A. p.^**^
**15.**	10.11.IIIA.	*M. furfur*	*M. furfur*	KC152897	*M. furfur* M235	100%	KC415088	*M. furfur* VG Ig 02	99%	*M. furfur*
**16.**	10.11.IV.	*M. sympodialis*	*M. sympodialis*	KC152901	*M. sympodialis* CBS 7222	100%	KC415091	*M. sympodialis* IFM 48109	100%	*M. sympodialis*
**17.**	45.08.II.	*M. furfur*	*M. furfur*	KC141972	*M. furfur* M235	100%	KC415099	*M. furfur* VG Ig 02	100%	*M. furfur*
**18.**	45.08.III.	*M. furfur*	*M. furfur*	KC141973	*M. sympodialis* CBS 7222	100%	KC415100	*M. sympodialis* IFM 48109	99%	*M. furfur* + *M. sympodialis*
**19.**	20.09.IV.	*M. furfur*	*M. furfur + M. sympodialis*	KC141965	*M furfur* M235	100%	KC415101	*M. furfur* VG Ig 02	99%	*M. furfur* + *M. sympodialis*
**20.**	17.09.IV.	*M. sympodialis*	*M. sympodialis*	KC109788	*M. sympodialis* MA 73	100%	KC415102	*M. sympodialis* IFM 48109	100%	*M. sympodialis*
**21.**	43.08.II.	*M. furfur*	*M. furfur*	KC141971	*M. furfur* M235	100%	KC415103	*M. furfur* VG Ig 02	100%	*M. furfur*
**22.**	6.11.IV.	*M. sympodialis*	*M. sympodialis*	KC152892	*M. sympodialis* MA 73	100%	KC415082	*M. sympodialis* IFM 48109	100%	*M. sympodialis*
**23.**	1.11.IA.	*M. globosa*	*M. globosa*	KC152884	*M. globosa* CBS 7966	96%	KC415074	*M. sympodialis* IFM 48109	100%	*M. globosa*
**24.**	1.11.II.	*M. globosa*	*M. globosa + M. restricta*	KC152885	*M. restricta* MRE28	99%	KC415075	*M. sympodialis* IFM 48109	100%	*M. globosa* + *M. restricta*
**25.**	1.11.IIIA.	*M. sympodialis*	*M. sympodialis*	KC152886	*M. sympodialis* MA 73	100%	KC415076	*M. sympodialis* IFM 48109	100%	*M. sympodialis*
**26.**	1.11.IVA.	*M. furfur*	*M. sympodialis*	KC152888	*M. sympodialis* MA 73	100%	KC415078	*M. sympodialis* IFM 48109	100%	*M. sympodialis*
**27.**	69.09.III.	*M. slooffiae*	*M. slooffiae*	KC141978	*M. slooffiae* CBS 7956	99%	KC415104	*M. slooffiae* sc7LG	99%	*M. slooffiae*
**28.**	69.09.IV.	*M. slooffiae*	*M. slooffiae*	KC141979	*M. slooffiae* CBS 7956	99%	KC415105	*M. sympodialis* IFM 48109	100%	*M. slooffiae* + *M. sympodialis*
**29.**	67.09.III.	*M. sympodialis*	*M. sympodialis*	KC141974	*M. sympodialis* CBS 7222	100%	KC415106	*M. sympodialis* IFM 48109	100%	*M. sympodialis*
**30.**	67.09.IV.	*M. slooffiae*	*M. sympodialis*	KC141975	*M. sympodialis* CBS 7222	100%	KC415107	*M. sympodialis* IFM 48109	100%	*M. sympodialis*
**31.**	68.09.III.	*M. sympodialis*	*M. sympodialis*	KC141976	*M. sympodialis* CBS 7222	100%	KC415108	*M. sympodialis* IFM 48109	100%	*M. sympodialis*
**32.**	68.09.IV.	*M. sympodialis*	*M. sympodialis*	KC141977	*M. sympodialis* CBS 7222	100%	KC415109	*M. sympodialis* IFM 48109	100%	*M. sympodialis*
**33.**	2.11.III.	*M. sympodialis*	*M. sympodialis*	KC152890	*M. sympodialis* MA 73	100%	KC415080	*M. sympodialis* IFM 48109	100%	*M. sympodialis*
**34.**	2.11.IV.	*M. obtusa*	*M. sympodialis*	KC152891	*M. sympodialis* MA 73	100%	KC415081	*M. sympodialis* IFM 48109	100%	*M. sympodialis*
**35.**	12.09.IV.	*M. sympodialis*	*M. sympodialis*	JX915741	*M. sympodialis* MA 73	100%	KC415110	*M. sympodialis* IFM 48109	100%	*M. sympodialis*

^*a*^Identification algorithm included macro- and microscopic examination of fungal colonies along with biochemical tests, as described in the Materials and Methods section.

^*b*^Based on two PCR-RFLP assays (i. e. with *Alu*I and *Hae*II restriction endonucleases).

^*c*^Results of BLAST search of GenBank (http://blast.ncbi.nlm.nih.gov/); The ITS and D1/D2 accession numbers are those under which the respective sequences determined in this study can be found in the GenBank; Percentage similarities with ITS or D1/D2 sequences deposited in the GenBank are given.

^*d*^Based on combined molecular methods results; ^*^C. d., *Cryptococcus diffluens*; ^**^A. p., *Aureobasidium pullulans*.

### Statistical analysis

The Chi-square test and Fisher’s exact test were used to evaluate the differences in the frequency and distribution of *Malassezia* species between the AD patients, PS patients, and healthy subjects. A *P* value < 0.05 was considered statistically significant.

### Ethics

The study was approved by the Ethics Committee of the Jagiellonian University in Kraków. All the patients gave informed consent to participate in the study.

## Results

A total of 35 *Malassezia* sp. cultures were obtained from clinical samples, with back being the most frequent site of isolation (16 cultures; 45.7% of all *Malassezia* cultures), followed by chest (10; 28.6%), face (5; 14.3%), and head (4; 11.4%). Of 24 skin samples collected from each of the three subject groups, 13 (54.2%), 12 (50%), and 10 (41.7%) gave positive culture in the control group, AD group, and PS group, respectively. The overall positive culture rate of the *Malassezia* yeasts from 4 different body sites of 18 patients under the study was 48.6% (35 positive samples out of 72 samples tested).

### Phenotypic identification

Based on conventional, phenotypic methods, all 35 yeast cultures were initially separated into 5 different *Malassezia* species. There were recognized 19 (54.3%) *M. sympodialis*, 7 (20%) *M. furfur*, 5 (14.3%) *M. slooffiae*, 2 (5.7%) *M. globosa*, and 2 (5.7%) *M. obtusa* isolates. Among the isolates from AD patients and healthy volunteers, *M. sympodialis* predominated and accounted for 66.7% (8/12) and 46.1% (6/13) of the isolates, respectively. In both these groups there were isolates of other four (control group) or three (all but *M. globosa*) species (AD group). In psoriatic patient group, only two species, equally abundant, were identified, namely *M. sympodialis* and *M. furfur*.

### PCR-RFLP analysis

PCR amplification of the partial 26S rRNA gene produced, for all isolates, a single amplicon of the expected size of *ca*. 550 bp. Upon digestion of the amplified products with the *Hae*II restriction enzyme 4 different restriction patterns could be distinguished. Three of them matched exactly the *Hae*II restriction patterns predicted for *M. sympodialis* (26 isolates; 74.3% of all isolates), *M. furfur* or *M. slooffiae* (6; 17.1%), and *M. globosa* or *M. restricta* (2; 5.7%). For one isolate (20.09.IV.) a mixed *Hae*II restriction pattern was obtained, corresponding to *M. sympodialis* and *M. furfur* or *M. slooffiae*.

A single amplicon of *ca*. 400–500 bp in size was generated by PCR amplification of the ITS2 region for all *Malassezia* isolates except one (20.09.IV.), for which two PCR products were visualized (one of *ca*. 400 bp and another of *ca*. 500 bp). When the PCR products were digested with the *Alu*I restriction enzyme 6 different restriction patterns were demonstrated, 4 of which corresponded to those expected for *M. sympodialis* (24 isolates; 68.6% of all isolates), *M. furfur* (4; 11.4%), *M. slooffiae* (2; 5.7%), and *M. globosa* (1; 2.9%). The restriction pattern of one isolate (1.11.II.) was a combination of those for *M. globosa* and *M. restricta*. The restriction patterns of 2 isolates (10.11.II.; 8.11.III.) contained a fragment characteristic of *M. sympodialis*, along with two additional fragments whose sizes did not correlate with the sizes of the *Alu*I restriction fragments previously reported for *Malassezia* species. In case of one isolate, for which two PCR products were obtained, they were both purified by gel extraction and subjected to *Alu*I digestion separately (20.09.IV.A/B). This resulted in 2 single restriction profiles, conforming to that of *M. sympodialis* and of *M. furfur*.

Based on the results from two PCR-RFLP assays, 31 (88.6%) of the isolates tested could be separated into 4 distinct *Malassezia* species, namely, *M. sympodialis* (24 isolates; 68.6% of all isolates), *M. furfur* (4; 11.4%), *M. slooffiae* (2; 5.7%), and *M. globosa* (1; 2.9%). Two isolates were identified as a mixture of two different *Malassezia* species, that is *M. sympodialis* and *M. furfur* (20.09.IV.) and *M. globosa* and *M. restricta* (1.11.II.). Finally, 2 isolates (10.11.II.; 8.11.III.) appeared to represent *M. sympodialis* mixed with another unknown fungal species.

### PCR-sequence analysis

The rDNA region containing the ITS1/ITS2 sequences and D1/D2 domains of the 26S rRNA was successfully amplified, for all isolates tested, resulting in a sole product of *ca*. 1,500 bp. The purified PCR products were used as templates in two independent sequencing reactions, targeting the ITS and D1/D2 loci, respectively. Upon ITS1/ITS2 sequence analysis, 33 (94.3%) of the isolates tested, could be unambiguously identified at the species level. The consensus ITS sequences of those isolates shared ≥99% similarity with the sequences of previously characterized fungal species, as evidenced by the BLAST search of the GenBank database. Twenty-four (68.6% of all isolates) isolates had a 100% similarity with the sequence of *M. sympodialis*. Four (11.4%) isolates showed 100% sequence identity with *M. furfur*, and 2 (5.7%) isolates showed 99% sequence similarity with *M. slooffiae*. The ITS1/ITS2 sequences of 2 isolates designated 8.11.III and 10.11.II., formerly identified as *M. sympodialis* (on PCR-RFLP analysis) showed a complete match (100% identity) to the ITS1/ITS2 sequences of *Cryptococcus diffluens* and *Aureobasidium proteae/pullulans*), respectively. The results for those two isolates were consistent, even if sequencing was performed on PCR products from different PCR runs using DNA from three different isolations. Sequence analysis of the ITS regions from one isolate (1.11.II.), identified as a mixture of *M. globosa* and *M. restricta* by PCR-RFLP analysis, was informative only after subcloning of the PCR product into the pGEM-T plasmid. Although several recombinants were examined, each time the ITS sequence homologous (99% identity) to that of *M. restricta* was resolved. Interestingly, when applying the same procedure (i. e. sequence analysis from the pGEM-T vector) to the second isolate (20.09.IV.) that was identified as consisting of two different *Malassezia* species (*M. furfur* and *M. sympodialis*), only a sequence with 100% similarity with that of *M. furfur* could be revealed. Two isolates designated 2.11.III. and 1.11.IA. yielded ITS1/ITS2 sequences with <99% similarity with the closest sequence in the GenBank database; the former gave the closest sequence match to *M. sympodialis*, whereas the latter – to *M. globosa*, at a similarity level of 97% and 96%, respectively (Table 2).

Sequence analysis of partial 26S rRNA gene allowed clear species discrimination of all isolates tested. The sequences of 33 (94.3%) isolates displayed ≥99% sequence identity to 4 *Malassezia* species strains, namely *M. sympodialis* (26 isolates; 74.3% of all isolates), *M. furfur* (4; 11.4%), *M. globosa* (2; 5.7%), and *M. slooffiae* (1; 2.9%). Finally, two isolates had a perfect match (100% sequence identity), one for *C. diffluens*, and the other for *A. pullulans*.

The results from the sequence analysis of the D1/D2 and ITS loci were almost entirely concordant, with the concordance rate, calculated as percent agreement between paired results, of 94.3% (33/35 cases). The only discrepant results were from 2 isolates designated 69.09.IV. and 1.11.II., identified by D1/D2 sequencing as *M. sympodialis* and *M. globosa*, respectively. Whereas the former isolate had previously been identified (both by PCR-RFLP analysis and ITS sequencing) as *M. slooffiae*, the latter had initially been recognized as a mixture of *M. globosa* and *M. restricta* (upon PCR-RFLP analysis), with the presence of only *M. restricta* confirmed by ITS sequencing. Based on the combined ITS and D1/D2 sequence analysis, those 2 isolates were considered to represent mixtures of two *Malassezia* species, that is *M. slooffiae* and *M. sympodialis* (69.09.IV.) and *M. globosa* and *M. restricta* (1.11.II.).

### Comparison of PCR-RFLP analysis and PCR-sequencing analysis

Concordance of the species identification results by using PCR-RFLP analysis and PCR-sequencing analysis was 85.7% (30/35 cases). Two isolates identified, upon PCR-RFLP analysis, as *M. sympodialis* (8.11.III.; 10.11.II.), were identified as *C. diffluens* (8.11.III.) and *A. pullulans* (10.11.II.) with PCR-sequencing. Another 2 isolates initially recognized as *M. furfur* (45.08.III.) and *M. slooffiae* (69.09.IV.) were re-identified as *M. sympodialis* and a mixture of *M. slooffiae* and *M. sympodialis*, accordingly. For one isolate (20.09.IV.) being a mixture of *M. furfur* and *M. sympodialis*, as demonstrated by PCR-RFLP, only the presence of *M. furfur* was confirmed by sequence analysis.

### Comparison of phenotypic and molecular methods

The results of phenotypic and molecular identification methods showed a concordance rate of 65.7% (23/35 cases). Twelve (34.3%) isolates produced discrepant results. These included 6 *M. sympodialis* isolates, which by phenotypic methods had initially been identified as *M. slooffiae* (in 3 cases), *M. obtusa* (2 cases), and *M. furfur* (one case), as well as 6 mixed-species isolates. Of the latter, morphological and biochemical tests correctly identified one of 2 co-occurring species in 5 cases. One isolate recognized as a mixture of *M. sympodialis* and *C. diffluens* had previously been identified as *M. furfur*, based on the physiological testing.

Overall, among the isolates under investigation, 29 (82.9%) were identified as homogenous species, namely *M. sympodialis* (24; 68.6%), *M. furfur* (3; 8.6%), *M. globosa* (1; 2.9%), and *M. slooffiae* (1; 2.9%). The remaining 6 (17.1%) isolates were demonstrated heterogeneous, being mixtures of two species; 2 isolates consisted of *M. furfur* and *M. sympodialis*, (20.09.IV.; 45.08.III.), one isolate contained *M. sympodialis* and *M. slooffiae* (69.09.IV.), and the other one *M. globosa* and *M. restricta* (1.11.II.). Two isolates were mixtures of *M. sympodialis* and a non-*Malassezia* species, that is either *C. diffluens* (8.11.III.) or *A. pullulans* (10.11.II.).

### Drug susceptibility testing

Evaluation of drug susceptibility of *Malassezia* spp. to 6 antifungal drugs was conducted on 19 isolates representing all *Malassezia* isolates cultured from 4 patients with AD, 4 patients with PS, and 4 healthy volunteers, randomly selected within each subject groups. All isolates examined were found susceptible to all 6 antifungals tested (Table 3). Six (31.6%) isolates showed intermediate susceptibility to at least one drug; 2 isolates (one of *M. furfur* (45.08.II.) and the other of *M. sympodialis* (12.09.IV.)) were intermediately susceptible to ECZ; one isolate, identified as a mixture of *M. slooffiae* and *M. sympodialis* (69.09.IV.) was intermediately susceptible to MNZ. Two isolates, identified as mixtures of *M. furfur* and *M. sympodialis*, and designated 45.08.III. and 20.09.IV. were intermediately susceptible to 2 (KTZ + ECZ) and 3 (KTZ + ECZ + MNZ) drugs, respectively. A three-drug intermediate susceptible pattern (KTZ + ECZ + MNZ) was also observed in one *M. furfur* isolate (43.08.II.). Overall, based on the inhibition zone diameter values, the more susceptible isolates included *M. slooffiae* and most of the *M. sympodialis* isolates, whereas the least susceptible isolates were represented by *M. furfur* and *M. furfur* co-cultured with *M. sympodialis* (Table 3).

**Table 3:** **Results of drug susceptibility testing of 19 *Malassezia *** **sp. isolates, as determined by Neo-Sensitabs assay**

***Malassezia *** **sp. (no.)**	**Antifungal drug**^***a***^											
	**CPO**		**FLZ**		**KTZ**		**ITZ**		**ECZ**		**MNZ**	
	**Zone [mm]**^***b***^	**Cat. (no.)**^***c***^	**Zone [mm]**	**Cat.(no.)**	**Zone [mm]**	**Cat. (no.)**	**Zone [mm]**	**Cat. (no.)**	**Zone [mm]**	**Cat. (no.)**	**Zone [mm]**	**Cat. (no.)**
*M. sympodialis* (13)	28	S (13)	43	S (13)	33.9	S (13)	36.5	S (13)	24.5	S (12)	26.5	S (13)
									17	I (1)		
*M. furfur* (2)	23	S (2)	36	S (2)	30	S (1)	29	S (2)	15.5	I (2)	20	S (1)
					25	I (1)						
											17	I (1)
*M. furfur* + *M. sympodialis* (2)	22.5	S (2)	39	S (2)	25.5	I (2)	28.5	S (2)	14.5	I (2)	20	S (1)
											15	I (1)
*M. slooffiae* (1)	30	S (1)	45	S (1)	35	S (1)	45	S (1)	30	S (1)	20	S (1)
*M. slooffiae* + *M. sympodialis* (1)	30	S (1)	40	S (1)	40	S (1)	40	S (1)	30	S (1)	16.5	I (1)

^*a*^CPO, ciclopirox; FLZ, fluconazole; KTZ, ketoconazole; ITZ, itraconazole; ECZ, econazole; MNZ, miconazole; number of isolates are given in brackets.

^*b*^Inhibition zone diameter mean value [mm] (each isolated was tested twice).

^*c*^Susceptibility categorization (S, susceptible; I, intermediate; R, resistant) based on the following criteria: CPO, ECZ, MNZ: S, ≥ 20; I, 12–19; R, ≤ 11; FLZ: S, ≥ 22; I, 15–21; R, ≤ 14; ITZ: S, ≥ 16; I, 10–15; R, < 10; KTZ: S, ≥ 30; I, 23–29; R, ≤ 22.

### Distribution of *Malassezia* species

The final species identification, based on the molecular data, was used to determine the frequency distribution of *Malassezia* species among different subject groups. Of the culture-positive samples from AD patients, all yielded *M. sympodialis* isolates. These were homogeneous cultures in all cases, except one, where a mixed culture of *M. sympodialis* and *C. diffluens* was obtained. Among the PS patients, 2 *Malassezia* species were identified, with *M. sympodialis* having been isolated from 7 (out of 9) samples of 5 (out of 6) patients, and *M. furfur* – from 5 samples of 4 patients. Those 2 species co-occurred in 2 samples from 2 different patients. A specimen from one psoriatic patient yielded a co-culture of *M. sympodialis* and *A. pullulans*. In the control group *M. sympodialis* was the most frequently observed, having been isolated from a total of 10 (out of 13) skin samples of all healthy subjects, including every sample of 4 of them. *Malassezia globosa* and *M. slooffiae* were isolated from 2 samples of single subjects either alone or in combination with *M. restricta* and *M. sympodialis*, respectively.

Overall, *M. sympodialis* was the predominant species in all 3 subject groups, having been cultured from a total of 29 (82.9%) skin samples collected from all subjects under the study except one PS patient. The second most common species was *M. furfur*, recovered from 5 specimens of 4 PS patients. *Malassezia globosa* and *M. slooffiae* were isolated from 2 samples, each, originating from 2 normal individuals. Two non-*Malassezia* species, namely *C. diffluens* and *A. pullulans* , both co-occurring with *M. sympodialis*, were recovered from a patient with AD and a patient with PS, respectively.

Statistical analysis of the data showed that the only significant association was that isolation of *M. sympodialis* alone was more frequent among AD patients and healthy volunteers, as opposed to PS patients, for whom isolation of other *Malassezia* and non-*Malassezia* species, either alone or mixed (also with *M. sympodialis*) was reported (*P <* 0.03).

## Discussion

Although several *Malassezia* species have been associated with various dermatological diseases, the exact pathological role of individual species remains obscured. An essential and still open question is whether there exists a relationship between particular *Malassezia* species and various skin disorders. Other clinical questions to be resolved include whether any of the *Malassezia* species preferentially occupies certain sites of the body, whether there are any differences in the distribution of the yeasts between lesioned and normal-appearing skin of patients, between adult and children, or between patients and healthy individuals, and finally whether there is variation in the prevalence of *Malassezia* species depending on gender, age, or geographical origin of the human host. There are now a growing number of works addressing these issues. For instance, studies of the *Malassezia* microbiota in healthy individuals consistently indicated *M. globosa* and *M. restricta* as the predominant species, with a combined detection rate of over 50% (in most of the studies) [7,31,32,34,35,49-51]. Whereas the involvement of the *Malassezia* species in SD and PV has been quite well recognized [35,50,52-58], the clinical role of these fungi in AD and PS is still controversial. The *Malassezia* yeasts are currently considered as contributory factors to the induction and exacerbation of both these conditions. The present study was to determine the composition of *Malassezia* microbiota on the skin of patients with AD and PS, and healthy volunteers from Poland. The choice of the study population was driven by two facts. First, there are much less data on the *Malassezia* microflora in AD and PS, than in other *Malassezia*-related diseases (i.e. PV, and SD). Second, AD and PS are the two most common chronic skin diseases, whose incidence has been on the rise in recent years in Poland. Among the Polish population, the prevalence of AD is around 1.4% in adults, and thrice as much (4.7%) in children aged between 3 to 16 years (but up to 32% in infants and young children, that is aged between 0–6 years) [59,60]. Psoriasis, on the other hand, is estimated to affect up to 1 million people in Poland (*ca*. 2.6% of general population) [61].

The *Malassezia* yeasts were cultured from almost half (48.6%) of the harvested skin samples, with the back being most heavily colonized body site, accounting for *ca*. 46% of the *Malassezia* cultures. The predominant *Malassezia* species in two clinical groups and the healthy control group was *M. sympodialis*. The recovery rate for this species among AD patients, PS patients, and healthy subjects was 100%, 70%, and 76.9%, respectively, and the overall recovery rate for *M. sympodialis* was 82.9%. The finding of such a high prevalence of *M. sympodialis* was rather unexpected, since other *Malassezia* species have usually been much more abundant in all three aforementioned groups, as reported by other authors. In a study of Nakabayashi *et al*., *M. sympodialis* was only the third most common species in lesional skin of AD Japanese patients (7% of samples), following *M. furfur* (21%), and *M. globosa* (14%) [50]. In a study from Sweden, *M. sympodialis* was absent from lesional sites of AD patients, whereas other *Malassezia* species (i. e. *M. globosa*, *M. obtusa*, *M. restricta*, and *M. slooffiae*) occurred at low rates of 3-11% [62]. Two further studies that used culture-independent, DNA-based methods for the detection of *Malassezia* species, showed *M. globosa* and *M. restricta* as the predominant species in AD. They were detected at frequencies ranging from 87.5% to 100%, while *M. sympodialis* at 40.6% and 58.3% [35,51]. However, in a Canadian study of Gupta *et al*., it was *M. sympodialis* that predominated in AD patients, with a detection rate of 51.5% [53]. Likewise, *M. sympodialis* was the dominant species among Korean AD patients, yet the isolation rate was low (16.3%) [33]. Among very few reports on the prevalence and species composition of *Malassezia* yeasts in PS patients, two were almost completely negative for the presence of *M. sympodialis*; in a study from Bosnia and Herzegovina, the predominant species in PS patients was *M. globosa* (55%) followed by *M. slooffiae* (17%) and *M. restricta* (10%) [63], while in a study from Iran, *M. globosa*, as the commonest species in PS (47%), was followed by *M. furfur* (39%) and *M. restricta* (11%) [64]. Similar data were obtained from a Japanese, culture-independent study of Takahata *et al*., who found *M. globosa* and *M. restricta* as the sole two *Malassezia* species in psoriatic scale samples, with similarly high detection frequencies of 98% and 92%, respectively [38]. However, in another study from Japan, as well as in a Canadian, culture-based study, *M. sympodialis* was found the third (50%), after *M. restricta* (91%) and *M. globosa* (68%) or the second (31%), after *M. globosa* (58%) most frequently isolated *Malassezia* species from psoriatic lesions [53,65]. As for the healthy skin, none of the hitherto performed studies have shown the predominance of *M. sympodialis*, as that seen in the present work. This species has usually ranked third in overall abundance among *Malassezia* species colonizing normal human skin, with the detection rate spanned from 10% to *ca*. 40% [7,31,32,35,50]. Two major components of the *Malassezia* biota of healthy individuals, that is *M. globosa* and *M. restricta* were seriously underrepresented in the current study, with an overall isolation rate of 15.4% and 7.7%, respectively. The disparities between the studies, as discussed above, in frequencies of *Malassezia* species isolations from different dermatological affections and body sites may be attributable to several factors, including geographical and ethnic origin, clinical and demographic characteristics and even lifestyle habits of the subjects under the study, but also certain methodological issues, such as the use of different sampling techniques (swabbing, scraping) or culture media (modified Dixon agar, Leeming-Notman agar). However, the distribution of *Malassezia* species is probably most influenced by a method used for species identification. Although the traditional identification schemes, based on morphological characteristics and biochemical activities, are in many clinical laboratories the only diagnostic methods available, they suffer from apparent limitations. Phenotypic tests are time-consuming, labour-intensive and often produce variable or inconclusive results, especially for newly described species; the final result relies on subjective interpretation by a laboratory expert. These methods are thus successively being complemented or replaced by DNA-based molecular techniques, of which PCR-RFLP analysis and PCR sequencing have most extensively been used [18,26,29-34,39-41]. Conceptually and technically PCR sequencing is the simplest. It is also the fastest and the most specific identification approach. An important advantage of DNA sequencing over PCR-RFLP is that the latter often involves a lengthy and laborious analysis of complex banding patterns, not always leading to a conclusive result. Moreover, DNA sequencing possesses a much higher discriminatory capacity, allowing intraspecies polymorphisms to be revealed. Some authors have already reported on the rDNA sequence heterogeneity within various *Malassezia* species, proving the existence of several individual genotypes within the species [26,39,40]. The intra-specific genetic diversity was also evidenced in this study. Two types of ITS sequences for *M. sympodialis* and two types of D1/2 sequences for *M. globosa* were demonstrated. Given the ability of DNA sequencing for strain typing and its potential use in phylogenetic and population genetics studies, along with the costs of sequencing rapidly plummeting, the method may soon become an integral part not only of a species identification algorithm but also of a routine epidemiological investigation.

The phenotypic and molecular identification results were discordant in *ca*. 35% of cases (12/35). The reason for this might either be the misidentification or the co-occurrence of different *Malassezia* species in one culture. The latter explanation is even more likely, as in the third of the discrepant cases, molecular methods did not invalidate the presence of a species identified by conventional phenotypic approach but only uncovered another, co-occurring *Malassezia* species. This in turn relates to the fact that the establishment of an axenic culture of a *Malassezia* species, uncontaminated by other *Malassezia* and non-*Malassezia* yeasts is rather challenging. A mixture of *Malassezia* species may not only be present in a clinical sample but even in a seemingly pure single colony on a culture medium. The co-isolation of two *Malassezia* species from the same specimen was recorded in 4 (11.4%) cases evaluated in this study. A higher prevalence of mixed *Malassezia* cultures was reported by other authors. For instance, in a Korean study of Lee *et al*., 15% of SD patients and 21% of healthy volunteers showed co-colonization of two or more *Malassezia* species [34]. In two European studies, *M. globosa*, the most commonly observed species, was associated in culture with other *Malassezia* species in 18% of PV patients from Greece [52] and 40% of PV patients from Spain [55]. Interestingly, in two cases in this study, *M. sympodialis* was co-cultured with a non-*Malassezia* species, namely *Cryptococcus diffluens* in an AD patient and *Aureobasidium pullulans* in a PS patient. Finding of *C. diffluens* on AD-affected skin is perfectly in line with previous research demonstrating this species to be a frequent colonizer of the skin surface of AD patients [66]. As for *A. pullulans*, it is a ubiquitous dematiaceous fungus that has emerged as an opportunistic human pathogen, especially among immunocompromised patients; it is a frequently isolated skin contaminant but rarely a causative agent of fungemia, systemic infections and abscesses in different viscera [67]. The discordance between phenotypic and molecular identification may also relate to the growth rate of different *Malassezia* species. It means that in a mixed culture, the fast-growing species, such as *M. sympodialis* may conceal the presence of the slow growers, such as *M. globosa*, *M. restrica*, or *M. obtusa*. This is also a possible explanation for the overall high frequency of *M. sympodialis* isolations in this study. Against this possibility is the fact that always a selected single separated colony from a primary culture served as an inoculum for the culture used for species identification tests. The six mixed-species cultures represent the only cases in which a primary culture colony already contained a mixture of distinct yeast species. Nevertheless, it seems that a molecular-based but culture-independent method would serve as a more accurate and reliable approach for the assessment of the diversity of *Malassezia* microbiota [28,35,38,51]. The use of culture-based approach for characterizing *Malassezia* communities from skin samples posed a limitation to the present study. Due to the fastidious nature of *Malassezia* fungi and difficulties in culture arising therefrom, the obtained results may understate the size and complexity (species structure) of the *Malassezia* microbiota.

Culture-based methods essentially select for those species that readily grow under the typical nutritional and physiological conditions supported by commonly used artificial media. These species may not represent the most abundant or influential organisms within a given locality [68].

There is a paucity of reports on drug resistance in *Malassezia* spp., and this mainly stems from the lack of a standardized protocol for *Malassezia* susceptibility testing. The observed variability of the results from laboratory to laboratory precludes any association of *in vitro* and *in vivo* responses of *Malassezia* yeasts to antifungals. In this study, the Neo-Sensitabs tablet diffusion assay was employed to test the susceptibilities of selected *Malassezia* strains to six drugs most widely used in the treatment of *Malassezia* infections, that is the azoles (FLZ, ECZ, ITZ, KTZ, and MNZ) and cyclopiroxolamine (CPO). All the analysed isolates were susceptible to those compounds, albeit the triazoles (FLZ and ITZ) and CPO were found to be more active than the azole derivatives (ECZ, MNZ, and KTZ). Noteworthy, strains of *M. furfur* generally appeared less susceptible than strains of *M. slooffiae* or *M. sympodialis*, indicating that certain *Malassezia* species develop mechanisms of drug tolerance more easily than other species do. Some variations between different *Malassezia* species in the susceptibilities to antifungal agents were also recorded by other authors [69,70]. Although the molecular bases of drug resistance in *Malassezia* fungi are largely unknown, there is an optimism that this will change now with an increasing body of data from the whole genome sequencing projects [71,72]. Recently, Kim *et al*. have demonstrated that genetic alterations in the amino acid sequence of a putative lanosterol 14α-demethylase (CYP51) from *M. globosa* may be responsible for resistance to azoles by blocking substrate access channels of the enzyme [73].

## Conclusions

To conclude, this study provides an important insight into the species composition of *Malassezia* microbiota in human skin. The most important findings can be summarized in three points. First, *M. sympodialis* was the predominant species in both normal and pathologic (AD- and PS-affected) skin. Furthermore, AD patients yielded exclusively *M. sympodialis* isolates, whereas isolates of *M. furfur* were observed only in PS patients. Whether this mirrors any predilection of particular *Malassezia* species for certain clinical conditions, and whether the overall dominance of *M. sympodialis* reflects geographical specificity needs to be evaluated on a much larger scale. Second, although the overall concordance between phenotypic and molecular methods was quite high (65%), with the discordant results being rather due to the presence of multiple species in a single culture (co-colonization) than true misidentification, for the identification of *Malassezia* species, molecular typing approach is preferred, as its results are more reliable and straightforward. Third, all *Malassezia* isolates were susceptible to cyclopiroxolamine and azole drugs, with *M. furfur* isolates being somewhat more drug tolerant than other *Malassezia* species.

## Abbreviations

AD: Atopic dermatitis; AFLP: Amplified fragment length polymorphism; BSA: Body surface area; CHS2: Chitin synthase; CLSI: Clinical and Laboratory Standards Institute; CPO: Ciclopirox; DGGE: Denaturing gradient gel electrophoresis; ECZ: Econazole; FLZ: Fluconazole; ITS: Internal transcribed spacer; ITZ: Itraconazole; KTZ: Ketoconazole; MDA: Modified Dixon’s agar; MEE: Multilocus enzyme electrophoresis; MNZ: Miconazole; NCBI: National Center for Biotechnology Information; PASI: Psoriasis area and severity; PCR-RFLP: PCR-based restriction fragment length polymorphism; PCR-SSCP: PCR-based single strand confirmation polymorphism; PFGE: Pulsed-field gel electrophoresis; PS: Psoriasis; PV: Pityriasis versicolor; RPB1: RNA polymerase subunit 1; SCORAD: Severity scoring of atopic dermatitis; SD: Seborrheic dermatitis.

## Competing interests

The authors declare that they have no competing interests.

## Authors’ contributions

TJ participated in the design of the study, supervised all experimental procedures, and wrote the entire manuscript. ER collected samples, performed drug susceptibility testing, participated in the design of the study and species identification. AZ performed the sequencing analyses. KR did the PCR-RFLP assays. AM and JB revised the manuscript critically for important intellectual content. All authors read and approved the final manuscript.

## Pre-publication history

The pre-publication history for this paper can be accessed here:

http://www.biomedcentral.com/1471-5945/14/3/prepub
